# Automated Conduction Velocity Analysis in the Electrohysterogram for Prediction of Imminent Delivery: A Preliminary Study

**DOI:** 10.1155/2013/627976

**Published:** 2013-12-29

**Authors:** Hinke de Lau, Chiara Rabotti, Rianne Bijloo, Michael Johannes Rooijakkers, Massimo Mischi, S. Guid Oei

**Affiliations:** ^1^Maxima Medical Center, P.O. Box 7777, 5500 MB Veldhoven, The Netherlands; ^2^Department of Electrical Engineering, University of Technology Eindhoven, Eindhoven, The Netherlands

## Abstract

*Background*. Analysis of the electrohysterogram (EHG) is a promising diagnostic tool for preterm delivery. For the introduction in the clinical practice, analysis of the EHG should be reliable and automated to guarantee reproducibility. *Study Goal*. Investigating the feasibility of automated analysis of the EHG conduction velocity (CV) for detecting imminent delivery. *Materials and Methods*. Twenty-two patients presenting with uterine contractions (7 preterm) were included. An EHG was obtained noninvasively using a 64-channel high-density electrode grid. Contractions were selected based on the estimated intrauterine pressure derived from the EHG, the tocodynamometer, and maternal perception. Within the selected contractions, the CV vector was identified in two dimensions. *Results*. Nine patients delivered within 24 hours and were classified as a labor group. 64 contractions were analyzed; the average amplitude of the CV vector was significantly higher for the labor group, 8.65 cm/s ± 1.90, compared to the nonlabor group, 5.30 cm/s ± 1.47 (*P* < 0.01). *Conclusion*. The amplitude of the CV is a promising parameter for predicting imminent (preterm) delivery. Automated estimation of this parameter from the EHG signal is feasible and should be regarded as an important prerequisite for future clinical studies and applications.

## 1. Introduction

Preterm delivery, defined as delivery before 37 weeks of gestation, constitutes a major problem in terms of neonatal mortality, morbidity, and healthcare costs [1–3]. Timely intervention and treatment with tocolytics and corticosteroids improves neonatal outcome [4]. However, the diagnostics currently used lack both sensitivity and specificity leading to both under- and overtreatment [5, 6]. A potential new diagnostic tool is the electrohysterogram (EHG), which is a noninvasive abdominal measurement of the electrical activity underlying uterine contractions.

The sequence of contraction and relaxation of the uterus results from a cyclic depolarization and repolarization of its smooth muscle cells in the form of action potentials (APs). APs occur in bursts; they arise in cells that act as pacemakers and propagate from cell to cell through gap junctions [7–9]. Labor and delivery are preceded by two physiological phenomena: increased excitability and increased connectivity among the cells, resulting in increased propagation of APs and more synchronized firing [10]. These changes are reflected in the recorded EHG.

The previous literature demonstrated that the EHG has great potential for monitoring labor, predicting labor time, and discriminating between physiological uterine activity and contractions leading to (preterm) delivery. Therefore, analysis of the EHG can support timely treatment of preterm labor [11–16]. To this end, several studies have focused on analyzing the spectral content of the EHG using either the peak frequency of the power density spectrum [15, 17–19] or the ratio between high and low frequency bands [11]. Another parameter from the EHG that has been proposed for predicting imminent preterm labor is the nonlinear correlation among channels in a multichannel recording [16].

Prior to delivery, the increased connectivity among cells also increases propagation, which can be assessed by estimating the conduction velocity (CV) from the EHG [20–22]. Differently from skeletal muscles, which are striated and present an anatomical direction of propagation parallel to the fiber orientation, the direction of propagation of the uterine APs is a priori unknown [23, 24]. Due to lack of evidence [25], many authors also concluded that no classical linear propagation of single APs could be assumed for the uterus and that only global propagation of the whole burst envelop could be measured [23, 25]. However, more recently, measurements of the electrical activity of the guinea pig uterus using a grid of extracellular electrodes clearly demonstrated that also for the uterus, similarly to the myocardium, a linear propagation of single APs can be measured [26]. However, direction and speed of AP propagation can change even within the same bursts.

Previous research mainly focused on the methods for measuring the CV [27–31]. Recently, the prognostic value of the CV for predicting imminent preterm delivery was investigated by visual inspection of the EHG signal [15]. Despite the very promising results presented in this clinical study, the employed visual approach has the disadvantage of not being reproducible. For use as a clinical tool, it would be desirable to rely on a fully automated CV analysis.

However, automated CV analysis entails a number of scientific challenges, namely, automatic detection of contractions, estimation of amplitude and direction of the CV vector, and exclusion of signals that are not related to propagating APs.

This study investigates the feasibility of a new automated approach for the analysis of the EHG CV for detecting imminent delivery. Our approach integrates previously validated EHG-based methods for contraction detection and automated analysis of the CV in two dimensions using a high-density electrode grid.

## 2. Materials and Methods

### 2.1. Study Protocol

A prospective observational cohort study was performed at the Maxima Medical Center Veldhoven, The Netherlands. Approval from the local medical ethical board was obtained and all the included women provided written informed consent for study participation. Patients with singleton pregnancies were enrolled, presenting with at least 3 contractions in 30 minutes, which were either perceived by the patient or visible on the external tocogram. Both term patients (gestational age 37 + 0*‒*41 + 6) and preterm patients (gestational age 24 + 0–36 + 6) were included. Exclusion criteria were oxytocin or prostaglandin administration prior to or during the measurement, induction of labor within 24 hours after the measurement, and known uterine malformation.

**Figure 1:**
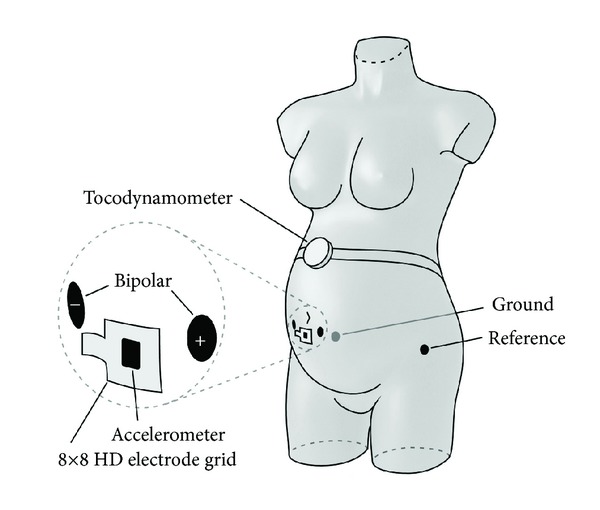
Measurement setup showing the position of all abdominal sensors.

Measurements from all enrolled patients were obtained using a measurement setup as shown in Figure 1. A 64-channel high-density (HD) electrode grid with external reference electrode on the hip was used in conjunction with a bipolar electrode pair (1 cm diameter, variable interelectrode distance) to record the EHG. Due to the a priori unknown AP direction of propagation, the bidimensional arrangement of the electrodes on the grid (8 × 8) permits estimating all the possible CV directions along the abdominal plane parallel to the abdominal surface. The bipolar signal initially used to allow recording other signals (such as the fetal ECG) was eventually employed to derive the contraction timing and trigger the CV vector estimation. The HD electrode grid could also be suitable, but the larger surface of the bipolar sensors offered better results for this specific purpose.

The recording of these signals was performed using a Refa multichannel amplifier (TMS International, Enschede, The Netherlands), with a patient ground on the hip. Simultaneously, a tocodynamometer was used as reference for contraction detection. For the same reason, the time instants at which the patient felt a contraction were annotated.

### 2.2. Signal Analysis

Here, a synthetic overview of the methodology used for the analysis is given. For further details, we refer to [31, 32].

The CV vector was identified during the contraction periods. Differently from previous studies, where contractions were annotated manually, we automatically derived an initial estimation of onset and duration of contractions. To this end, an estimate of the internal uterine pressure (IUP) was derived from the bipolar EHG signal. Based on a validated method [32], indicated by *n* and *f*, the discrete time and frequency variables, respectively, the unnormalized first statistical moment Ψ(*n*) of the bipolar EHG spectrogram, *ρ*(*n*, *f*), was calculated in a selected frequency band, [*f*_min⁡_, *f*_max⁡_]; that is,

(1) $$\begin{array}{c} \Psi \left(n\right)=\overset{f_{\max}}{\underset{f=f_{\min}}{\sum }}f\rho \left(n,f\right), \end{array}$$

with *f*_min⁡_ = 0,3 Hz and *f*_max⁡_ = 0, 8 Hz. An adaptive threshold was then used to detect onset and duration of each contraction in 60 s overlapping windows [33]. Differently from our previous work [3], no modeling was used to improve the estimation accuracy of the IUP amplitude. A more accurate estimation of the IUP, which was out of the scope of the present work, would not significantly improve the accuracy of the thresholding procedure used to assess onset and duration of contractions.

Of the contractions selected by EHG signal analysis, only those that were visible on the external tocogram or concurred with annotations of contractions as felt by the patient were eventually selected for further analysis.

**Figure 2:**
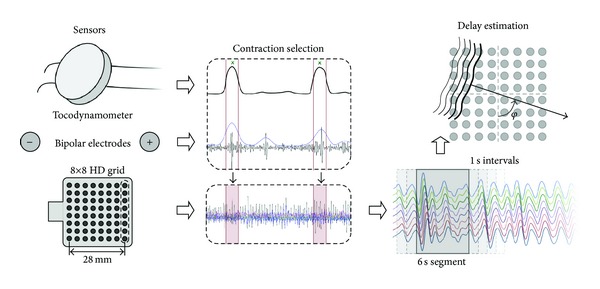
The method for analyzing the CV. The EHG is recorded using a 8 × 8 HD electrode grid plus a bipolar electrode pair. Contractions are selected based on the eIUP, which is derived from the bipolar electrodes, plus the external tocodynamometer and maternal perception. Finally, the delays are estimated in overlapping windows using a maximum likelihood approach.

In the signal segments selected as contractions, the CV vector was identified in two dimensions from the 8 × 8 HD electrode grid in overlapping segments (5 s overlap). Following the schematic representation of Figure 2, we describe the EHG propagation by a CV vector *v*. The vector has an amplitude and an incidence angle *θ*  (*θ* ∈ [−*ππ*]) with respect to the vertical axis of the electrode grid. The signal is detected by *N*_*r*_ rows and *N*_*c*_ columns of electrodes. Assuming that the same signal shape *s*(*n*) is measured at each channel, the signal *x*_*r*,*c*_ measured at the channel (*r*, *c*) in the *r*th row and *c*th column of the electrode grid can be modeled as

(2) $$\begin{array}{c} x_{rc}\left(n\right)=s\left(n-\left(r-1\right)\tau_{r}-\left(c-1\right)\tau_{c}\right)+w_{r,c}\left(n\right), \end{array}$$

where *n* indicates the time sample (*n* = [1, 2,…, *N*]) and *w*_*r*,*c*_(*n*) is the white Gaussian noise which is present at the channel (*r*, *c*). As from (2), we assume the linear propagation of the APs; that is, in each channel (*r*, *c*), the reference signal shape *s*(*n*) is delayed by *τ*_*r*_ and *τ*_*c*_ time samples relative to the previous row and column, respectively. Identification of the vector *v* requires estimation of (*τ*_*r*_, *τ*_*c*_), which we obtain using a maximum likelihood approach, that is, by maximization of the probability density function *p*((*τ*_*r*_, *τ*_*c*_) | *x*_*rc*_(*n*), *s*(*n*)) in the frequency domain, where *τ*_*r*_ and *τ*_*c*_ can be estimated without resolution limits. Under the assumption of white Gaussian noise, the maximum likelihood approach is equivalent to the minimization of the cost function *E* ^2^ defined as

(3) E2(τr,τc) =2N∑r=1Nr∑c=1Nc∑f=0(N/2)−1[Xrc(f)−S(f)e−j2πf[(r−1)τr−(c−1)τc]],

indicated by *f*, the discrete frequency; *X*_*rc*_(*f*) and *S*(*f*) are the Fourier transforms of the signal recorded at the channel (*r*, *c*) and of the reference shape, respectively. Following the description in Figure 2, for an interelectrode distance equal to *d*, it follows that *τ*_*r*_ and *τ*_*c*_ are related to the CV amplitude and to the incidence angle *θ* by

(4) $$\begin{array}{c} \tau_{r}=\frac{d\cos\theta }{\text{CV}},\\ \tau_{c}=\frac{d\sin\theta }{\text{CV}}. \end{array}$$

The use of different weighting strategies of the derived cost function was introduced in [31] to deal with poor interchannel signal similarity due to the presence of noise. The weights are inversely proportional to the estimated channel noise. Of the different weighting strategies proposed in [31], we chose the weighted cost function with the best estimation accuracy.

Segments with a calculated CV value above 30 cm/s, which are significantly higher than the physiological values reported in the literature [23, 26], were considered as outliers and were excluded.

### 2.3. Statistical Analysis

Patients delivering within 24 hours after the measurement were classified as labor group and those delivering outside this time window were classified as nonlabor group. CV and propagation path were compared between these groups. In order to be independent of the number of analyzed segments and contractions per patient, an average CV vector was identified for each analyzed contraction and subsequently the average CV vector for each patient was determined. The Shapiro-Wilk test was used to test for a normal distribution of the estimated values of CV vector amplitude. Levene's test was applied to test for equal variances in the labor and nonlabor groups. Finally, an independent samples *t*-test was used to test for a significant difference in amplitude of the CV between both groups. The alpha was set to 0.05 for all statistical tests.

## 3. Results

**Figure 3:**
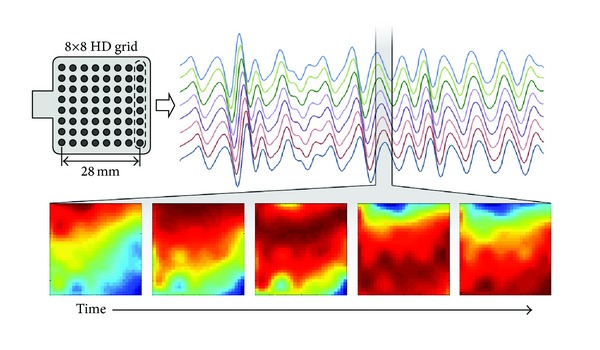
Next to the HD electrode grid, the EHG signals of eight electrodes (one column) are shown during a contraction. The five images at the bottom show an interpolated 2D representation of a single EHG pulse propagating from top to bottom.

Twenty-two patients were included in the study, of which 7 were preterm. Nine patients delivered within 24 hours and were classified as labor group.  Table 1 shows the baseline characteristics of the labor and nonlabor groups. An example of a downward propagating wave of uterine activity during a contraction visualized by the adopted high-density grid of 64 electrodes can be seen in Figure 3.

**Figure 4:**
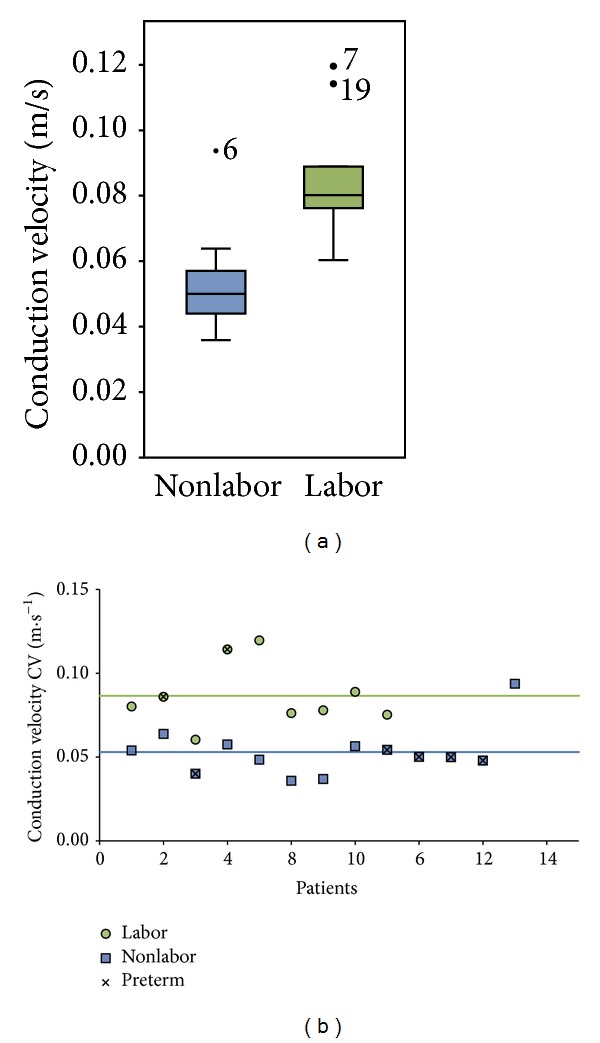
On the left, a boxplot shows the average amplitude of the CV vector for the labor and nonlabor groups. The median value is displayed with a horizontal line. On the right, a scatter plot shows the individual average amplitudes of the CV vector for both groups. The horizontal lines represent the average value for the labor and nonlabor groups.

In total, 64 contractions were analyzed.  Figure 4 shows the boxplot of the mean CV for the patients in the labor and the nonlabor groups; the median values of the CV of the groups as a whole are indicated as a horizontal line. The Shapiro-Wilk test was insignificant, supporting the null hypothesis that the data is derived from a normally distributed population. Similarly, Levene's test showed an insignificant result, supporting equal variances in the labor and nonlabor groups. The average amplitude of the CV vector was significantly higher for the labor group, 8.65 cm/s ± 1.90, compared to the nonlabor group, 5.30 cm/s ± 1.47 (*P* < 0.01). The angle of propagation showed a high variability among patients in both of the labor and nonlabor groups, even within the same contraction.

## 4. Discussion

This study investigates the feasibility of a new automated approach for the analysis of the EHG CV for detecting imminent delivery. Our approach integrates validated EHG-based methods for contraction detection and automated analysis of the CV in two dimensions using a high-density electrode grid [31]. The patients in this study presented with uterine contractions and were classified as labor group or nonlabor group based on delivery within or after 24 hours, respectively. The results show a significantly higher amplitude of the CV vector in the labor group.

The measurements were performed in a diverse group of patients featuring both term and preterm patients admitted for varying reasons. The common denominator was that all patients had palpable and measurable contractions. The emphasis was placed on investigating the feasibility of automated CV analysis, in order to open the way to future clinical studies and applications based on this parameter as diagnostic tool for imminent (preterm) birth. The assumption here is that comparable changes in conduction properties can be observed in contractions leading to preterm and term delivery. In follow-up studies, it will be important to have a consistent group of patients presenting with premature contractions and who are considered for treatment with tocolytics based on gestational age and clinical parameters.

In this study, additional data other than the EHG signal was used for detecting contractions, namely, an external tocodynamometer and annotations of subjectively perceptible contractions by the patient. This was chosen to achieve a more robust distinction between uterine activity and measurement artifacts. In future work, a fully automated selection of contraction segments and analysis of CV should be pursued. However, while an automated method ensures reproducibility and should therefore be preferred for everyday clinical use, visual inspection might be required in a preliminary phase for discriminating uterine activity from noise and excluding artifacts and signals that do not propagate linearly, from the analysis.

Noteworthily, identification of the EHG CV vector using the present methods implies the assumption that the signal does propagate and that propagation is linear. While it is reasonable to hypothesize that the linearity of the propagation could be a discriminative parameter for predicting imminent delivery in itself, several aspects related to the evolution from pregnancy to labor are not yet fully understood and need further dedicated research [34]. Therefore, we excluded spikes propagating nonlinearly from the analysis as we expected those cases to be outliers, that is, to have a CV outside the physiological range reported by the previous literature [26, 35]. Only [15] reported values higher than 30 cm/s; these values cannot be considered as a physiological reference due to the specific measurement setup, which allows for information on only a projection of the CV vector [36].

Finally, another novelty of this study is the use of a 64-channel high-density electrode grid for recording the EHG. Due to the a priori unknown AP direction of propagation, the bidimensional arrangement of the electrodes on the grid permits estimating all the possible CV directions along the abdominal plane parallel to the abdominal surface. Furthermore, due to the grid dimensions, planar wave propagation could be assumed and the small interelectrode distance enables following the same spike (action potential) from one electrode to the other [29]. In the present study, we intended to use the conduction velocity as an independent predictor of imminent delivery reflecting the increased propagation of action potentials between myometrial cells. Therefore, we chose a high-density grid with relatively small dimension. However, uterine activity throughout the whole uterus might provide additional information on imminent delivery and for that purpose we would consider a larger grid preferable. Ideally, a combination of local propagation and global synchronicity should be pursued, and this will be possibly considered in our future studies. Moreover, in order to improve user friendliness and simplify signal analysis, a reduced number of electrodes could be used, and depending on the chosen electrode configuration, a different hypothesis (e.g., point source) may be considered for propagation.

## 5. Conclusion

In agreement with previous studies, our results show that the CV vector amplitude is a promising parameter for predicting imminent (preterm) delivery. Automated estimation of this parameter from the EHG signal is feasible and should be regarded as an important prerequisite for future clinical studies and applications in this context. Therefore, these results open the way to future studies on the accuracy of EHG parameters, such as the CV, for timely and accurate diagnosis of imminent preterm delivery.

## Figures and Tables

**Table 1:** Patient characteristics.

	Labor	Nonlabor
Number of patients	9	13
Gestational age (weeks + days)^1^	31 + 1–40 + 4 (37 + 2)	26 + 2–41 + 3 (36 + 1)
Preterm	2	5
Nulliparous	4	8
Age^1^	17–36 (27.9)	16–36 (27.8)
BMI^1^	22–42 (28.2)	24–34 (26.8)
Hours to delivery^1^	1–10 (6)	27–1488 (255)

^1^Mean value in parentheses.
